# Geological Carbon Sequestration: A New Approach for Near-Surface Assurance Monitoring

**DOI:** 10.3390/ijerph8030818

**Published:** 2011-03-11

**Authors:** Lucian Wielopolski

**Affiliations:** Environmental Science Department, Brookhaven National Laboratory, Bldg. 490, Upton, NY 11973, USA; E-Mail: lwielo@bnl.gov; Tel.: +1-631-344-3656

**Keywords:** carbon, monitoring, geological sequestration, spectroscopy, neutrons, gamma-rays, errors, minimum detectable limits

## Abstract

There are two distinct objectives in monitoring geological carbon sequestration (GCS): Deep monitoring of the reservoir’s integrity and plume movement and near-surface monitoring (NSM) to ensure public health and the safety of the environment. However, the minimum detection limits of the current instrumentation for NSM is too high for detecting weak signals that are embedded in the background levels of the natural variations, and the data obtained represents point measurements in space and time. A new approach for NSM, based on gamma-ray spectroscopy induced by inelastic neutron scatterings (INS), offers novel and unique characteristics providing the following: (1) High sensitivity with a reducible error of measurement and detection limits, and, (2) temporal- and spatial-integration of carbon in soil that results from underground CO_2_ seepage. Preliminary field results validated this approach showing carbon suppression of 14% in the first year and 7% in the second year. In addition the temporal behavior of the error propagation is presented and it is shown that for a signal at the level of the minimum detection level the error asymptotically approaches 47%.

## 1. Introduction

Global warming and climate change are attributed to increases in the concentration of greenhouse gases (GHG) in the atmosphere, from anthropogenic emissions of CO_2_, from the pre-industrial revolution level of about 260 ppm, to present day concentrations of about 391 ppm, viz., ∼35% increase [1]. The main sources of GHG emissions are associated with burning fossil fuels, changing land usage, and cultivation of the soil. To combat global climate change will require a combination of approaches including improving energy efficiency and using alternative energy sources. Predictions of the increased use of energy globally during this century and continued reliance on fossil fuels point to a further rise in GHG emissions [2] with a concomitant one in atmospheric CO_2_ concentrations. These consequences cannot be abated unless major changes are made in the way energy is produced and used; in particular, how carbon is managed [3,4]. Mitigating the forecast increase in fossil-fuel consumption includes producing clean fuels, capturing industrially generated CO_2_, and sequestering this CO_2_ in deep geologic formations (carbon capture & sequestration (CCS)). The attractiveness of the CCS program stimulated significant investments by governments and the private sector to develop the necessary technologies, and to evaluate whether CO_2_ control could be implemented safely and effectively to maintain the CO_2_ in reservoirs. The United States Department of Energy (USDOE) prepared a roadmap for the CCS program [5]. The program’s early planners recognized the potential risks of geological storage to humans and ecosystems that might arise from leaking injection wells, abandoned wells, across faults, and from ineffective confining layers. Hence, cost-effective, robust monitoring must be an integral part of and specifically designed for every individual CCS project.

**Figure 1.**
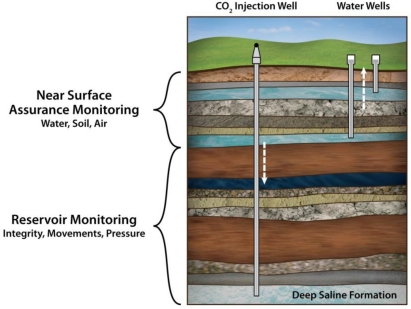
Scheme of two monitoring regions: One, near the surface for assurance monitoring; and, two, deep monitoring for evaluating the reservoir’s integrity.

Monitoring the status and the fate of a CO_2_ plume from geological carbon sequestration (GCS) projects is mandatory as stipulated by the Environmental Protection Agency’s (EPA’s) permitting processes for underground injections [6–9]. The monitoring generally falls into two types; monitoring deep reservoirs to confirm their stability and integrity, and, monitoring above the reservoir, *i.e.*, near-surface monitoring (NSM) of water, air, and soil to assure public health and environmental safety. The IPCC and the USDA reports outline these two domains, differing in their objectives and the instrumentation required for monitoring [10,11], as depicted schematically in Figure 1. In general, the IPCC guidelines [10] stipulate a 99% reservoir-retention capacity over a 1,000 year period. That, for a 200 Mt CO_2_ reservoir, translates into a yearly acceptable leak of 2,000 t/year or ∼5.5 t/day. Considering the surface area of a reservoir through which a gas could leak, its tortuous passage and dispersion on its movement from a depth of several thousand feet to the surface, we would expect very low fluxes of CO_2_ to be evident at the surface. The exceptions might be leaks occurring near injection- and abandoned-wells, or known geological faults. Many of the well-established techniques of monitoring CO_2_ in the atmosphere and in the near-surface areas were adopted directly for assessing leaks from geological carbon-sequestration sites in spite of their inadequate sensitivities and point measurements in space and time. Table 2 of the USDOE’s report summarizes their basic characteristics and the challenges they pose for detecting low-level signals [11]. Thus, current instrumentation faces a double challenge of reducing the minimum detectable limit (MDL) with minimum detectable change (MDC), and distinguishing real changes from natural ones due to seasonal- and diurnal-variations in the field CO_2_ fluxes. Point measurements might well be inadequate when the location of the leak is unknown, so that it probably is necessary to couple them with line- and area-integrated CO_2_ measurements, or design sensor networks to cover the area [12,13].

To address the hurdles of the MDL, field natural variability and point measurements, a new approach that, rather than directly measuring the fluxes of seeping CO_2_, measures a secondary quantity, namely total carbon in soil (TOC). Since the soil’s CO_2_ levels affect its pH and the activity of the plants’ roots it contains, they influence the TOC levels. Hence, a slow CO_2_ seepage will increase cumulatively the soil CO_2_ content inversely impacting TOC. Lower noise and reduced natural variability surrounding the TOC, lowering the MDL levels is enabled. Measurements of TOC offer a temporal- and spatial-integration of the impact of prolonged low seepage of CO_2_. Time integration is accomplished by measuring the cumulative effect on the TOC of prolonged exposure to changes in soil CO_2_ [14]; Wielopolski and Mitra earlier reported such a decrease in TOC [15]. Others detailed the overall degradation of vegetation caused by CO_2_ leaks from underground CO_2_ springs in Mammoth Mountain, California, and in Latera caldera, Italy [16,17]. This paper emphasizes the benefits of the error reduction of the proposed new system and of using unique scanning capacity of the inelastic neutron scattering (INS) system for spatially integrated monitoring. Thus, the hypothesis tested is that a CO_2_ leak would impact the vegetation and result in a near surface carbon suppression; like in the vicinity of natural CO_2_ vents; and the objectives are to demonstrate the validity of the hypothesis and suitability of the INS to measure these changes. INS system is briefly described and the reduction in the error propagation and lowering of the MDL and MDC are outlined. Theoretically, both can be reduced to reasonably low levels.

## 2. Site and Setup

### 2.1. Site

**Figure 2.**
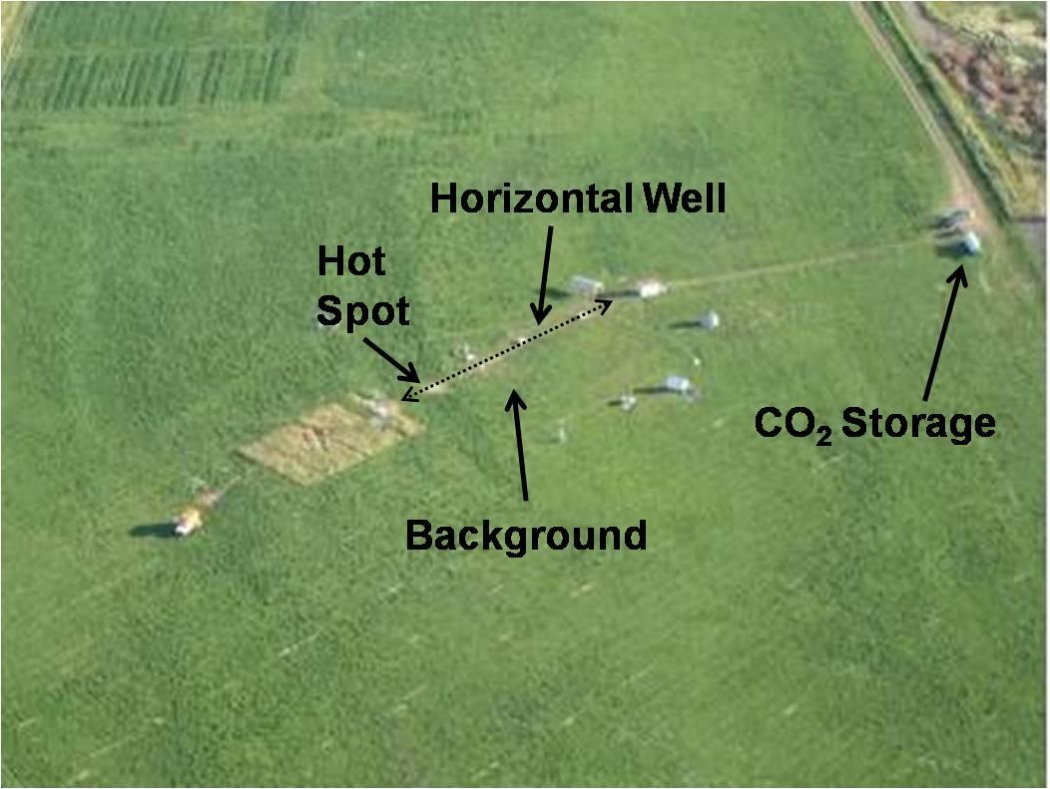
Site of the ZERT facility showing: a CO_2_ storage tank, a flow control hat, and the location of the horizontal well. It also shows the measurement sites over a hot spot and the background region.

The applicability of INS for monitoring GCS was demonstrated at the zero emission research and technology (ZERT) facility located on a former agricultural plot at the western edge of the Montana State University-Bozeman campus, Bozeman, Montana, USA. This facility was established for testing and tuning instrumentation for studying near-surface CO_2_ transport and detection under controlled conditions. The site, located at an elevation of 1,495 m, is covered with vegetation consisting primarily of alfalfa *(Medicago sativa)*, yellow blossom sweet clover *(Meliotus officinalis)*, dandelion *(Taraxacum officinale*, Canada thistle *(Cirdium arvense)*, and a variety of grasses (family Poaceae). The field is typical of the Bozeman area, with alluvial sandy gravel deposits overlain by a few meters of silts and clays with a blanket of topsoil. There are two distinct soil horizons; a topsoil, some 0.2 to 1.2 m thick, of organic silt, clay, and some sand, and an underlying deposit of sandy gravel extending down to about 5 m. Carbon-dioxide was introduced through a 100 m long horizontal well installed between 1 and 2.5 m deep, and injected at a rate of 0.3 tons per day for twenty eight days; Spangler *et al.*, give more detailed information on the site and injection system [18,19]. Figure 2 shows the site with the CO_2_ storage tank, and the transport line to a control hut that regulates and monitors the flow through the horizontal well. The hot spots indicate regions of high CO_2_ flow that degraded the vegetation.

### 2.2. INS System

**Figure 3.**
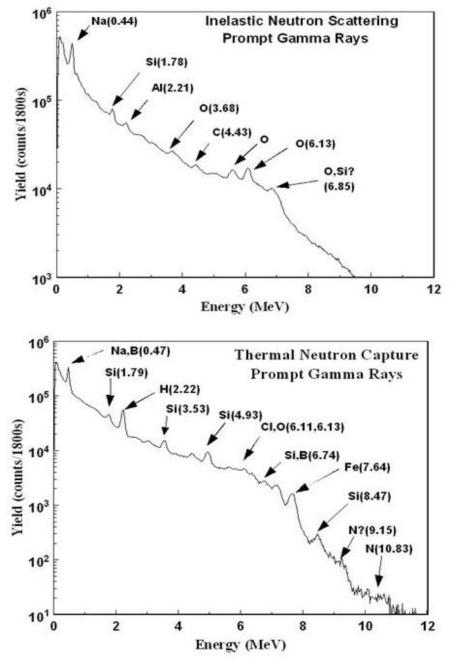
Typical gamma-ray spectra induced by inelastic neutron scattering and thermal neutron capture reactions.

The INS method is based on spectroscopy of gamma rays induced by fast (14 MeV) neutrons interacting with the elements present in soil via inelastic neutron scattering and thermal neutron capture processes. The INS system consists of a neutron generator (NG) that is turned off at the end of the data acquisition, detection and spectroscopy systems, and a power supply, all of which are mounted on a cart about 30 cm above the ground, thus enabling use in stationary or scanning modes of operation. Analysis and calibration of the characteristic elemental gamma-ray spectra resulting from inelastic neutron scatterings and thermal neutron captures (Figure 3) provide quantitative information on elemental concentrations in soil. The INS system interrogates large soil volume of about 0.3 m^3^ to an effective depth of ∼30 cm, as detailed by Wielopolski *et al.* [15,20]. The linear correlation between INS signal counts and carbon concentration was demonstrated in synthetic soils [21] and in natural fields using soil chemical analysis [22,23]. Thus, the net number of counts in the carbon peak can be expressed in terms of surface carbon concentration (g C/cm^2^) using the slope of a regression line. Similarly, INS system’s signal resulting from scanning capabilities, a key feature for spatial averaging, is converted to carbon content using the same calibration line. This is pertinent for detecting low level signals over large areas where the actual location of the leak is unknown. Uniquely, the error and MDL in the INS system can be lowered by extending the counting time or increasing the system’s sensitivity, *i.e.*, by increasing the number of detectors. These features are demonstrated in the following section on spectral analysis.

The soil carbon measurements at the ZERT facility were taken by placing the INS system above a .hot spot., marked in Figure 2 that was impacted by CO_2_ leakage from the horizontal well. These measurements were compared with those taken away from the horizontal well.

## 3. Spectral Analysis

**Figure 4.**
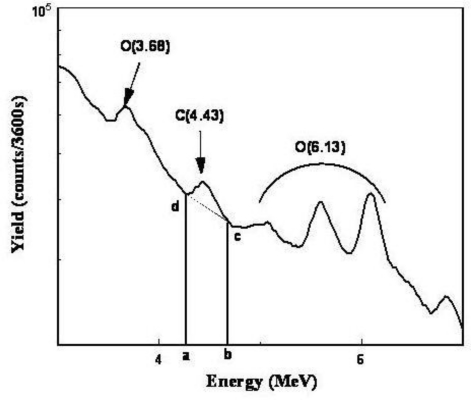
Partial gamma-ray spectrum in which an energy interval “ab”, located under the carbon photopeak, marks the boundary of the total counts, T_t_, and a background counts enclosed by the trapezoid’s area “abcd”.

Statistics of nuclear counting follows a binomial distribution, which for a large number of counts N > 12 can be approximated by a normal distribution with a mean value, N, and standard deviation (SD) the square-root of N (sqrt(N)) [24]. By extension, in nuclear spectroscopy, the gamma-ray events in the detector are represented by the number of counts falling into contiguous energy intervals (channels). Figure 4 depicts a partial spectrum with expanded energy intervals where interest lies with the number of counts in the energy interval ‘ab’ embracing a carbon peak. The total number of counts in that energy interval T_t_ following T minutes of counting time is due to unknown incident signal counting rate S_r_ times T, and the background counting rate B_r_ times T. Thus, T_t_ = S_r_T + B_r_T in which B_r_T is the area of a trapezoid ‘abcd’ marked in Figure 4. Conversely, the net number of counts associated with an element (E) of interest, S_r_T, is given by the difference T_t_ – B_r_T. The INS’s net counts are converted to conventional units of areal density (g E/m^2^) by dividing the net signal by the sensitivity of the system, s, defined as the number of counts acquired during a counting period T, S_r_T, per gram element per unit area; k is proportionality constant with matching units of g E/m^2^. Thus s = S_r_T/k, which also is the slope of the regression line that correlates INS yield *versus* the soil’s carbon concentration. The experimentally determined quantities B_r_, S_r_ and s represent the key performance parameters of an INS system from which other parameters are derived. Using the general uncertainty estimator of a function f(x,y,z…) given, to a first approximation, by Equation 1 [25],

(1) $$\sigma_{f}^{2}=\sum_{i}\left[\sigma_{i}^{2}{\left(\frac{\partial f}{\partial x}\right)}_{i}^{2}\right]$$

It is possible to derive the SD of S_r_T as σ_S_ = √(T_tot_ + B_r_T) = √((S_r_ + 2B_r_)T). The minimum detection limit (MDL) is defined as the number of counts above the background that differs from the background by a given confidence level; for example for a 99% confidence level the peak must contain three standard deviation counts above the background, and thus we can write:

(2) $$\text{MDL}=3\times \surd ({\text{B}}_{\text{r}}\times \text{T})\;\;\;(\text{counts})$$

Further, the relative SD for a signal at the MDL level, RSDMDL, is given by σ_MDL_/MDL_c_, Equation 3,

(3) $$\text{RSDMDL}=\surd [2/9+1/3\text{sqrt}({\text{B}}_{\text{r}}\text{T})]$$

**Figure 5.**
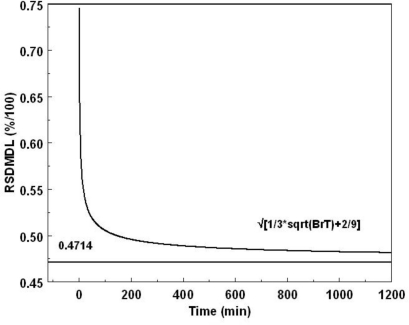
Relative standard deviation of a minimum detection limit signal (RSDMDL) based on Equation 3; it is bounded at 75%, and asymptotically approaches 47.14% for long counting times.

The RSDMDL, plotted in Figure 5, is bound between 0.745 for B_r_T = 1 and approaches asymptotically 0.471 for B_r_T→∞, B_r_ or T can be changed independently.

The elemental density corresponding to the number of counts given in Equation 2 is obtained by dividing Equation 2 by s, thus,

(4) $${\text{MDL}}_{\text{E}}=(3\times \text{k}/{\text{S}}_{\text{r}})\times \surd ({\text{B}}_{\text{r}}/\text{T})/\text{s}\;\;\;\;\;\;\;\;\;\;\;(\text{g}\;\text{E}/{\text{cm}}^{2})$$

Similarly, the minimum detectable change (MDC) defined as a change of three standard deviations in the signal level error, we can write,

(5) $$\text{MDC}=3\times \surd (({\text{S}}_{\text{r}}+2{\text{B}}_{\text{r}})\times \text{T})\;\;\;\;\;\;(\text{counts})$$

and, in terms of elemental concentration,

(6) $${\text{MDC}}_{\text{E}}=(3\times \text{k}/{\text{S}}_{\text{r}})\times \surd (\left({\text{S}}_{\text{r}}+2{\text{B}}_{\text{r}}\right)/\text{T})/\text{s}\;\;\;\;\;\;\;(\text{g}\;\;\text{E}/{\text{cm}}^{2})$$

**Figure 6.**
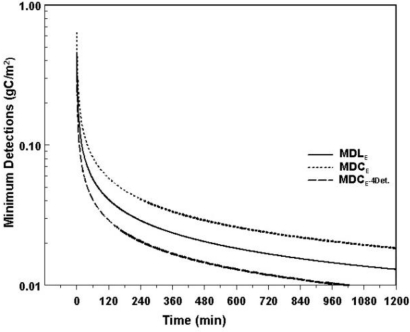
Minimum detection limit, MDL, and minimum detection change, MDC, respectively, based on Equations 4 and 6 *versus* counting time T. Increasing the sensitivity also impacts the MDC.

From Equations 4 and 6, it is apparent that increasing the counting time reduces the MDL_E_ and the MDC_E_. Similarly, increasing the sensitivity of s or S_r_, the signal counting-rate, by increasing the number of detectors also will lower the MDL_E_ and MDC_E_. Finally, reducing the background counting-rate by improving the shielding of the system also will lower MDL_E_ and MDC_E_. These features are graphed in Figure 6.

## 4. Results

Soil carbon measurements were taken over two 28-day injections episodes, in 2008 and in 2009. The soil carbon levels were measured above a HS pre- and post-injection and away from the horizontal well. No chemical analysis of soil samples were performed in order not to disturb the soil CO_2_ flow conditions. The net carbon yields, taken over one hour show a drop in soil carbon levels above a hot spot while simultaneously demonstrating no changes in silicon, oxygen and other elements in the background or above the HS; Table 1 shows the net counts in silicon (Si), oxygen (O) and carbon peaks [15]. To plot the graphs given in Equations 2, 4, and 6 the background count-rate, B_r_, was averaged over the two injection episodes, Table 2. The lower background in 2009 is attributed to the malfunctioning of one of the three detectors, thus reducing the background by about a third. Correcting for this anomaly in 2009, the estimated mean background rate, B_r_, was about 50,000 counts/min, and the sensitivity, s, was approximately 1,500 counts/min/(kg C/m^2^). Using these values the relative SD of a signal at the level of the detection limit given by Equation 3 is plotted *versus* time (Figure 5). Using the same values for B_r_ and s, the MDL_E_ and MDC_E_, were calculated using Equations 4 and 6, respectively, and plotted in Figure 6. Quadrupling the number of detectors quadruples the signal and the background reducing the MDL_E_ and MDC_E_ by a factor of two. This is shown by the graph MDL_E_-4Det in Figure 6.

## 5. Discussion

Ideally no underground leakage of CO_2_ should be occurring from underground reservoirs regardless of their size. However, practically, some very low leaks in the order of 0.01% over the expected life-time of a reservoir may be acceptable. The dispersion of the leaks over the reservoir’s surface area and their dilution during migration toward the surface would result in very low changes in the surface fluxes. These amounts are below the detection limits of the current instrumentation that was tuned at test facilities operating with higher fluxes. Furthermore, current instrumentation provides point measurements in time and space. At potential leak sites, this instrumentation is being used near injection- and old abandoned-wells, and possibly along known faults. The concerns with MDLs and with covering the entire area above the reservoir, which may amount to hundreds of square-kilometers, continually are addressed by developing new improved instrumentation. One new approach is to monitor secondary parameters that are affected by CO_2_ fluxes or, alternatively, combining a few modalities to improve the signal-to-noise ratio. Examples of secondary quantities include the quality of the drinking water, reflectance spectroscopy of the vegetation above-ground, and impact on the species forming the vegetation. However, noise levels and natural fluctuations continue to pose problems.

Monitoring carbon in soil, using an INS system, is yet another indirect method to detect possible leaks from deep reservoirs. The viability of INS was demonstrated by detecting a drop in the soil’s carbon levels following fumigation with CO_2_. The uniqueness of INS approach offers time integration of a cumulative effect of a low leak that slowly influences the vegetation and near-surface pH levels that, in turn, alter the carbon level. The non-destructive measurements made by INS enable us to acquire sequential readings in exactly the same spot. Its sensitivity is further enhanced by the ability to measure large volumes of soil when operating in static- and scanning-modes; in principle, this enables coverage of the entire area above the reservoir, thus providing spatial averaging of the signal from the entire site. These features are well suited for monitoring possible changes in the soil carbon for potential leaks in any location. In addition, a very unique feature of INS is that we can reduce errors and lower the detection limits by extending the counting time, increasing the sensitivity of the system, or lowering the background, thus enhancing the capacity of INS to detect potential CO_2_ leaks.

The elemental peaks shown in Table 1 do not exhibit the same drop in 2009 as does the background in Table 2. The reason for this is not completely clear. It is speculated that, since the background radiation is more multidirectional than the specific peaks that originate in the soil, this may have to do with geometric factors depending on which detector malfunctioned, viz., the middle one or one of the side detectors. More experiments are needed to clarify this difference in response, as are others to determine the threshold values at which CO_2_ fluxes begin to affect the vegetation and near-surface carbon storage.

## 6. Summary

The hypothesis that leaking CO_2_ suppresses the near surface carbon was validated and suitability of the INS system to measure these changes in soil was demonstrated. INS is a unique addition to the arsenal of tools for monitoring geological carbon sequestration. This new approach using INS offers the possibility of temporal-spatial integration, thus enhancing the capability for detecting low-level leaks. In addition, the paper detailed how the measurement error, MDL_E_ and MDC_E_, can be reduced by extending the counting time and increasing the system’s sensitivity. INS alone or in combination with other system will improve monitoring capabilities and enhance the success of the CCS programs. It would be highly desirable to perform controlled experiments in which soil CO_2_ levels are doubled and record the threshold levels impacting the vegetation and TOC. These would have to be performed with different soil types.

## Figures and Tables

**Table 1.** Analyses of the Si, O, and C peaks of the INS spectra measured during the 2008 and 2009 injection periods. Measurements were taken at a hot spot (HS), and the background (B) was determined in 2008 off the horizontal well; in 2009 it was determined off the well and at a pre-injection HS.

**2008—During Injection**						
	**Hot Spot (HS)**			**Background (B)**		
	Si	O	C	Si	O	C
**N**	8	8	7	12	12	11
**Mean**	1,031,332	622,914	47,137	1,008,545	636,929	53,704
**STD Deviations**	24,858	28,328	3,610	16,530	48,235	4,731
**STD Deviations (%)**	2.4	4.5	7.4	1.6	7.5	8.8
**STD Error (%)**	0.8	1.6	2.8	0.5	2.2	2.7
**Δ(1 − HS/B) × 100**	2.3	−2.2	−14.0	---	---	---
**2009—Pre-Injection**						
	**Hot Spot**			**Background**		
	Si	O	C	Si	O	C
**N**	9	9	8	5	5	5
**Mean**	787,977	650,746	79,728	759,986	665,833	81,228
**STD Deviations**	15,066	13,714	4,850	5,860	5,811	3,916
**STD Deviations (%)**	1.9	2.1	6.1	0.8	0.9	4.8
**STD Error (%)**	0.6	0.7	2.2	0.4	0.4	2.1
**Δ(1 − HS/B) × 100**	3.7	−2.3	−1.9	---	---	---
**2009—Post-Injection**						
	**Hot Spot**			**Background**		
	Si	O	C	Si	O	C
**N**	9	9	8	3	3	3
**Mean**	842,562	628,521	78,850	812,448	635,948	84,718
**STD Deviations**	19,889	7,117	6,079	5,751	8,725	4,566
**STD Deviations (%)**	2.4	1.1	7.6	0.7	1.4	5.4
**STD Error (%)**	0.8	0.4	2.7	0.4	0.8	3.1
**Δ(1 − HS/B) × 100**	3.7	−1.2	−6.9	---	---	---

**Table 2.** Mean background counts during 2008 and 2009, and combined over two years; n is the number of measurements, SDEV is the standard deviation, and CV is the coefficient of variation (SDEV/sqrt(n)).

**Year**	2008	2009	Combined
**n**	20	27	47
**Mean**	3,338,759	2,102,343	3,232,342
**SDEV (%)**	43,119 (1.29)	15,384 (0.73)	65,455 (2.03)
**CV (%)**	9,642 (0.29)	2,961 (0.14)	9,548 (0.30)
